# Temporal trends and inequalities in coronary angiography utilization in the management of non-ST-Elevation acute coronary syndromes in the U.S.

**DOI:** 10.1038/s41598-018-36504-y

**Published:** 2019-01-18

**Authors:** Muhammad Rashid, David L. Fischman, Martha Gulati, Khalid Tamman, Jessica Potts, Chun Shing Kwok, Joie Ensor, Ahmad Shoaib, Hossam Mansour, Azfar Zaman, Michael P. Savage, Mamas A. Mamas

**Affiliations:** 1 Keele Cardiovascular Research Group, Centre for Prognosis Research, Institutes of Applied Clinical Science and Primary Care and Health Sciences, Keele University, Stoke-on-Trent, UK; 2 Department of Cardiology, Royal Stoke Hospital, University Hospital North Midlands, Stoke-on-Trent, UK; 3 Department of Medicine (Cardiology), Thomas Jefferson University Hospital, Philadelphia, Pennsylvania United States; 4 Division of Cardiology, University of Arizona, Phoenix, AZ USA; 5 Department of Cardiology, International medical centre, Jeddah, Saudi Arabia; 6 Department of Cardiology, Aswan University, Aswan, Egypt; 7 Department of Cardiology, Freeman Hospital and Institute of Cellular Medicine, Newcastle University, Newcastle-upon-Tyne, UK

## Abstract

Coronary angiography (CA) is the basis of an invasive management strategy in non-ST elevation acute coronary syndromes (NSTEACS). There are limited contemporary data on national temporal trends in utilization of CA in different patient subgroups. We sought to investigate temporal trends, predictors and clinical outcomes associated with the use of CA in the US. Using the Nationwide Inpatient Sample (NIS) from 2004–2014, we identified all inpatient admissions, age ≥18, with a primary diagnosis of NSTEACS. Descriptive statistics and multivariable logistic regression models were used to investigate temporal trends, predictors and clinical outcomes associated with CA. From a total of 4,380,827 patients, 57.5% received CA during the study period and were more likely to be male, younger and less comorbid as defined per Charlson comorbidity index. The proportion of patients receiving CA increased from 48.5% to 68.5%, however, higher proportional increase was observed in males (53.9% to 69.4% P_trend_ < 0.001) and those age ≤60 years (59.0% to 77.9% P_trend_ < 0.001). Prior history of CABG (OR 0.33 95%CI 0.35–0.36), previous PCI (OR 0.84 95%CI 0.83–0.86) and previous AMI (OR 0.65 95%CI 0.64–0.67) were inversely related with receipt of CA. Receipt of CA was strongly associated with decreased odds of in-hospital mortality (OR 0.38 95%CI 0.36–0.40). In this national analysis, we observed a temporal increase in utilization of CA albeit slower adoption was noted in older, women and more comorbid patients. The risk-treatment paradox wherein patients who are most likely to benefit were less likely to receive CA persists even in contemporary practice.

## Introduction

Non-ST-Elevation acute coronary syndromes (NSTEACS) are estimated to account for almost two-thirds of total hospital admissions for an acute coronary syndrome in the United States and Europe^1–4^. Despite the use of pharmacoinvasive strategies, NSTEACS remains the most vulnerable acute coronary syndrome phenotype with high mortality and morbidity^5–8^. Guidelines from national bodies emphasize the use of coronary angiography (CA) in patients presenting with NSTEACS particularly in unstable or high-risk patients^9,10^ with data from observational and randomized control trials demonstrating improved outcomes in patients receiving early invasive CA^11,12^.

Despite the established benefit of an early invasive strategy in patients with NSTEACS, significant variations in the utilization of CA both at regional and national levels remain. The decision to undertake an invasive approach in the form of CA followed by revascularization requires careful consideration of the patient’s baseline risk profile and coexisting comorbidities^10,13^. However, there is limited data from contemporary national populations on how patient’s baseline risk profile and coexisting comorbidities have changed over time, and in particular how these may have influenced CA practice in the real world setting.

In this study, we used the National Inpatient Sample (NIS) database to explore the secular trends in utilization of CA in NSTEACS patients. We investigated if the receipt of CA in contemporary practice differs in patients stratified according to age, sex, ethnicity, and comorbidity burden and hospital characteristics. We also examined predictors of CA and the association between use of CA and clinical outcomes.

## Methods

### Study settings

NIS is the largest publically available all-payer inpatient healthcare database, developed by Healthcare Cost and Utilization Project (HCUP)^14^, which is sponsored by the Agency for Healthcare Research and Quality (AHRQ). NIS collects discharge level data from approximately 1000 hospitals, including 20% of all community hospital in the US, with over 7 million hospital admissions added each year. Discharge weights are used to determine national estimated and weighted data contains over 35 million hospital records truly representative of a large national sample. NIS is a publically available database with no identifiable patient information; therefore, ethical approval was not sought for this study.

### Study design

This is a retrospective analysis of all records in NIS dataset of patients admitted with a primary diagnosis of NSTEACS from 2004 to 2014. We used International Classification of Diseases, Ninth Revision, Clinical Modification (ICD-9-CM) code of 411.1 and 410.7 to identify all admissions with a primary diagnosis of NSTEACS, age >18 years. Further restrictions were applied to exclude records with missing data on age, sex, length of stay, median zip code income, in-hospital mortality, primary expected payer and total charges. This analysis included only emergency or urgent admissions excluding the elective admissions as they may not represent true diagnoses of NSTEACS. CA was defined as ICD-9-CM procedure codes 88.53, 88.54, 88.55, 88.56 37.22 and 37.23, with or without percutaneous coronary intervention (PCI) (ICD-9-CM procedure codes 00.66, 360.1, 360.2, 360.5, 360.6 and 360.7.

We collected data regarding patient baseline demographics including age, sex, race, primary expected payer, admission day of the week and cardiovascular risk factors (known coronary artery disease (CAD), family history of premature CAD, smoking, dyslipidaemias, previous myocardial infarction (MI), history of coronary artery bypass graft (CABG), previous percutaneous coronary intervention (PCI), previous stroke or transient ischemic attack) and chronic hypertension. The ICD-9-CM codes or clinical classification software codes used to identify any additional comorbidities are provided in Supplementary Table 1. Patient co-morbidity status was determined using information from 29 Elixhauser comorbidities as defined by AHRQ. Deyo modification of Charlson Co-morbidity Index (CCI) was derived using a point-based system with scores ranging from 1 to 6, with each value weighted depending on the prognostic impact of the comorbidity^15^. These scores were then summated to classify overall comorbidity burden into mild, moderate and severe categories with CCI score of 0,1,2 and 3 or more respectively (Supplementary Table 2). Finally, we collected information regarding the hospital characteristics including bed size, location, region and teaching status. The hospital bed size within NIS are defined using different regions of US and ranges from 1–249 beds for a small hospital, 25–449 for a medium hospital and 50+ to 450+ for a large size hospital.

Outcome of measures were in-hospital all-cause mortality, adverse cardiac complications, major bleeding, and vascular complications. Adverse cardiac complications were a composite of cardiac tamponade, pericardiocentesis, iatrogenic cardiac complication requiring emergency coronary artery bypass graft (CABG) surgery and hemopericardium. Major bleeding was a composite of gastrointestinal, retroperitoneal, intracranial or unspecified haemorrhage, and requirement of blood transfusion. Vascular complications were defined as procedure-related vascular injury. All complications were identified using ICD-9-CM codes in any of the secondary diagnosis fields within the NIS database (Supplementary Table 3).

### Statistical analysis

All statistical analyses were conducted using Stat 14.0 (College Station, Texas, USA). We employed descriptive statistics to compare differences in baseline demographics, hospital characteristic and crude outcome rates of patients who received CA compared to those managed medically. Continuous variables are reported as median and interquartile ranges to account for skewness of data. Categorical variables are presented as number and percentage. Chi-square and t-tests were used to determine statistical difference between patients who received CA compared to those who were managed medically for categorical or continuous variables respectively, while the “nptrend” package was used for trend across ordered groups. Univariable and multivariable analyses were undertaken to determine the association between use of CA and outcomes of interest. Logistic regression models were fitted using maximum likelihood estimation and were adjusted for all potential and measured confounders including age, sex, year of procedure, 29 Elixhasuer comorbidities, ethnicity, median income, weekend/weekday admission, cardiovascular risk factors and hospital characteristics. In order to better control for any differences in the baseline characteristics of the patients in the two groups, we also undertook a sensitivity analysis using propensity score matching. We estimated the average treatment effects using the “teffects” package and included all the variables as described in the multivariable logistic regression models.

## Results

### Patients and hospital characteristics

**Figure 1:**
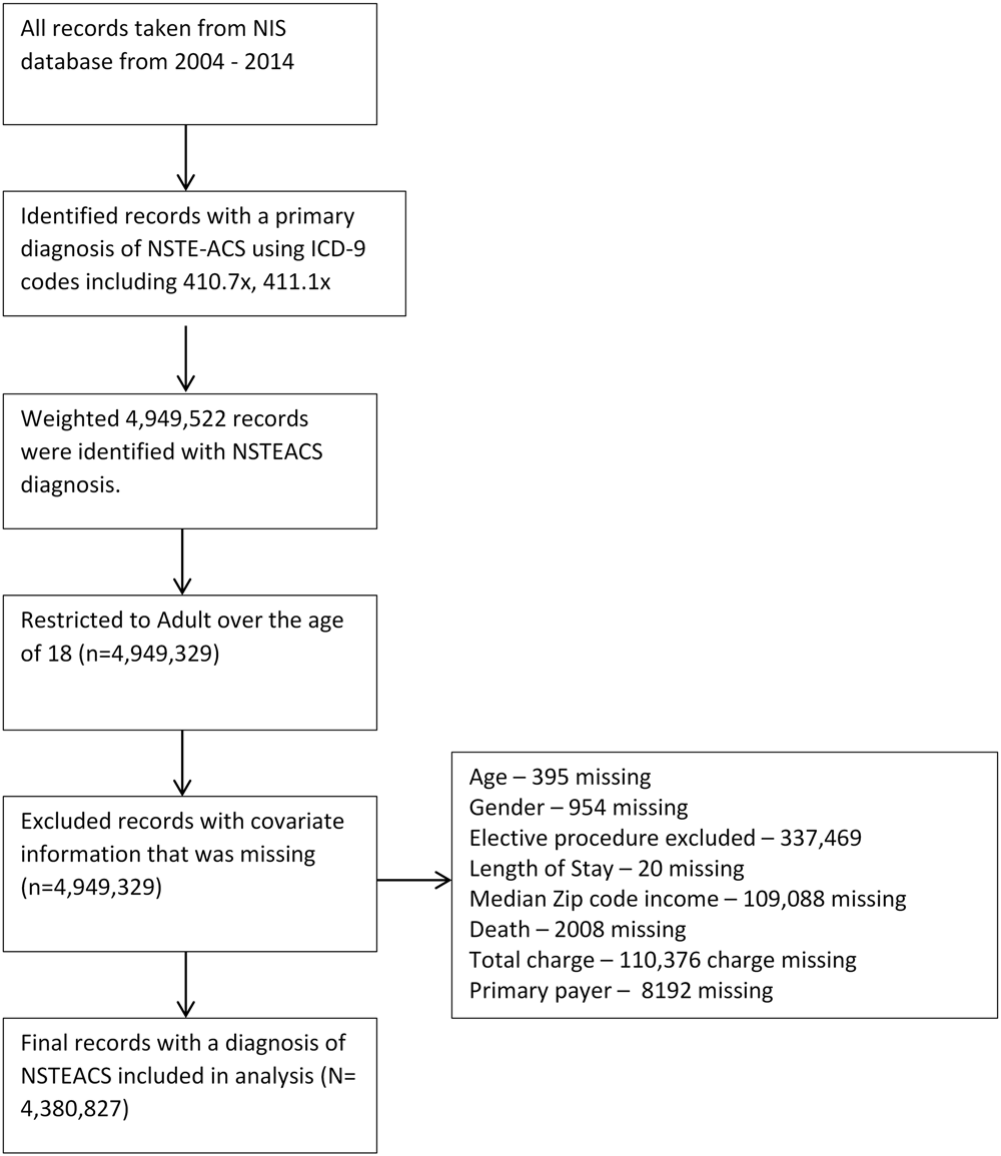
Flow diagram of included/excluded records.

A total of 4,380,827 patients were admitted with a diagnosis of NSTEACS between 2004 and 2014 of which 2,518,704 (57.5%) underwent an in-patient CA (Fig. 1). Differences in baseline characteristics between patients receiving CA compared to those managed medically are presented in Table 1. Patients receiving a CA were younger (median age 65 vs 72 years, p < 0.001), had worse cardiovascular profile such as history of smoking (37.9% vs 22.4%, p < 0.001), dyslipidaemia (56.4% vs 37.5%, p < 0.001), previous history of PCI (11.5% vs 7.7%, p < 0.001) and IHD (81.7% vs 42.6%, p < 0.001). Conversely, medically managed patients were more likely to be women (51.3% vs 39.3%, p < 0.001), had higher proportions of co-existing comorbidities (CCI ≥ 3 53.9% vs 46.1%, p < 0.001) and were more likely to be admitted on weekend days (26.8% vs 25.0%, p < 0.001).

**Table 1:** Baseline characteristics of patients receiving medical management compared to those receiving coronary angiogram.

	Patients receiving medical management	Patients receiving coronary angiogram
Number of Cases weighted (%age)	1,862,123 (42.5%)	2,518,704 (57.5%)
Age (year), Median IRQ	72 (63–85)	65 (46–75)
Men %	49.7%	61.7%
Ethnicity		
White	63.2%	63.3%
Black	10.0%	9.0%
Hispanic	6.4%	6.2%
Asian/Pacific Islander	1.8%	1.6%
Native American	0.4%	0.5%
Other	2.2%	2.7%
Missing Race	16.0%	16.7%
Weekend admission	26.8%	25.0%
Primary expected payer, %		
Medicare	72.2%	53.3%
Medicaid	5.7%	6.6%
Private Insurance	16.2%	30.3%
Self-pay	3.6%	6.2%
No charge	0.3%	0.7%
other	1.9%	2.9%
Median Household Income (percentile)		
0–25^th^	30.2%	29.2%
26–50^th^	27.0%	27.7%
51–75^th^	22.7%	23.9%
76–100^th^	20.1%	19.2%
Comorbidities, %		
Dyslipidaemia	37.5%	56.4%
Smoking	22.4%	37.9%
Previous AMI	9.5%	9.4%
History of IHD	42.6%	81.7%
Previous PCI	7.7%	11.5%
Previous CABG	10.1%	5.8%
Previous CVA	4.0%	3.1%
Family history of CAD	3.7%	8.0%
Valvular heart disease	0.4	0.1
Peripheral vascular disease	11.9%	11.9%
Use of assist devise or IABP	0.5%	4.2%
Shock	2.2%	2.6%
AIDS	0.12%	0.13%
Alcohol abuse	2.4%	2.9%
Deficiency anaemias	20.3%	13.2%
Chronic Blood loss anaemia	1.6%	0.8%
RA/collagen vascular		
diseases	2.4%	2.2%
Congestive heart failure	1.3%	0.5%
Chronic pulmonary disease	25.4%	20.6%
Coagulopathy	4.4%	4.2%
Depression	7.8%	6.8%
Diabetes	30.1%	30.3%
Diabetes with complications	7.5%	6.1%
Drug abuse	1.8%	2.2%
Hypertension	68.2%	70.9%
Hypothyroidism	12.6%	9.4%
Liver disease	1.4%	1.2%
Lymphomas	0.7%	0.4%
Fluid and electrolyte disturbances	25.1%	16.1%
Other neurological disorders	9.1%	4.0%
Obesity	9.1%	14.6%
Paralysis	2.6%	1.2%
Psychoses	2.7%	1.9%
Pulmonary circulation disorder	0.2%	0.06%
Renal failure (chronic)	24.8%	15.0%
Peptic ulcer disease	0.05%	0.04%
Weight loss	3.2%	1.5%
Solid tumor without mets	2.0%	1.1%
Metastatic cancer	1.5%	0.4%
Dementia	12.9%	2.5%
Charlson Comorbidity Index		
0	23.0%	34.5%
1	31.5%	32.8%
2	24.6%	19.5%
≥3	20.9%	13.1%
Hospital bed size		
Small	16.3%	8.0%
Medium	28.3%	21.7%
Large	55.4%	70.3%
Hospital Region		
Northeast	25.8%	17.9%
Midwest	20.1%	24.4%
South	38.8%	42.4%
West	15.2%	15.3%
Location/Teaching status		
Rural	18.0%	6.6%
Urban-non teaching	47.2%	39.0%
Urban- teaching	34.8%	54.4%
Length of stay, Median (IQR)	3 (2–6)	3 (2–6)
Total charge, $, Median (IQR)	18078 (9841–34417)	51433 (31694–85583)
Bleeding complications	11.9%	10.7%
Vascular complications	0.3%	1.4%
Cardiac complication	0.5%	2.1%
In-hospital mortality	6.6%	1.9%

Medically managed patients had higher crude in-hospital mortality (6.6% vs 1.9%, p < 0.001) and bleeding complications (11.9% vs 10.7%, p < 0.001). The median length of stay was similar in both groups (3[IQR 2–6] days) whereas receipt of CA was associated with greater hospital costs compared to medically managed patients. (Median total charge $51433[(IQR $31694-$85583] vs $18078[(IQR $9841-$34417]).

### Temporal Trends

**Figure 2:**
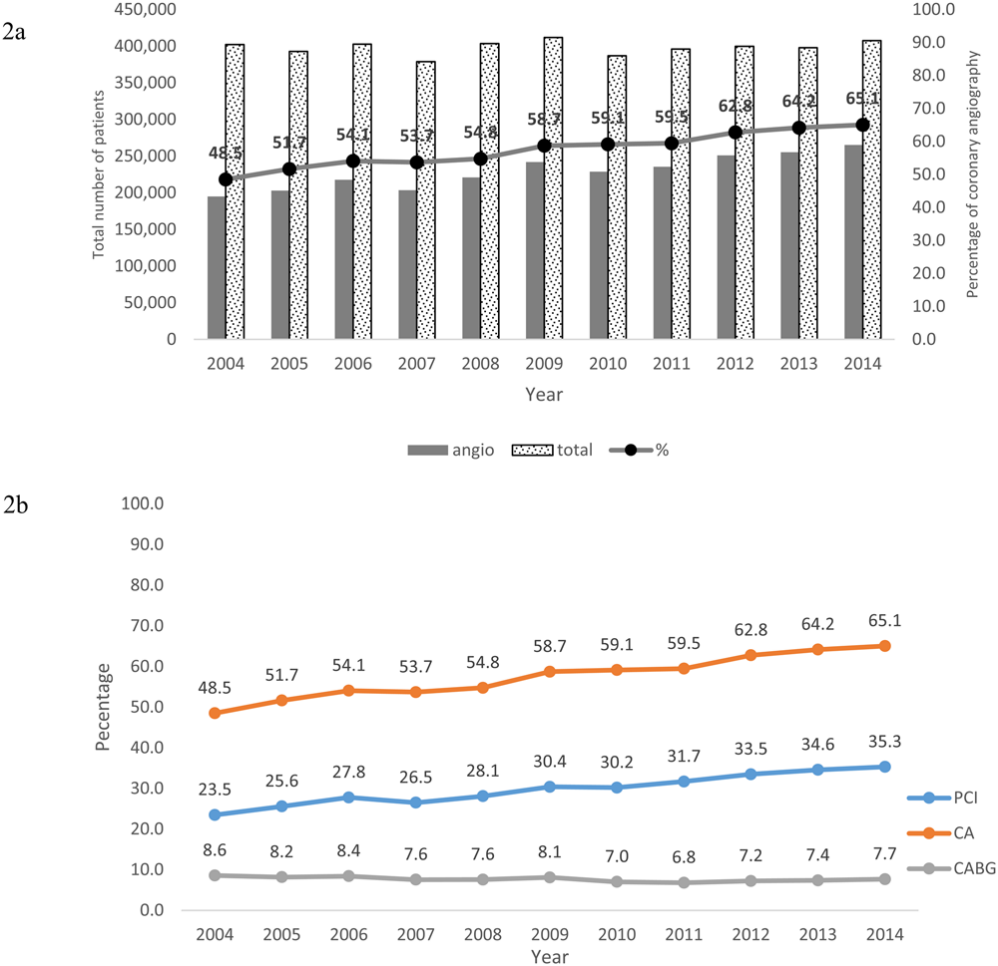
Temporal Trends in Use of Coronary angiography from 2004–2014.

Use of CA increased from 48.5% in 2004 to 65.1% (P_trend_ < 0.001) in 2014 (Fig. 2) which mirrored with increase in PCI activity in US and a concomitant steady decline in CABG procedures (decreased from 8.6% to 7.7%). Temporal changes in baseline demographics and hospital characteristics, comorbidities and crude outcomes in patients receiving CA and those who were medically managed are presented in Tables 2 and 3 respectively. There was greater proportional increase in the prevalence of risk factors for coronary artery disease including smoking (27.8% to 46.7%, P_trend_ < 0.001), dyslipidemia (46.7% to 61.8%, P_trend_ < 0.001), previous AMI (7.8% to 11.4%, P_trend_ < 0.001), hypertension (62.0% to 77.2%, P_trend_ < 0.001), and peripheral vascular disease (9.7% to 13.1%, P_trend_ < 0.001) in the CA group. Patients receiving CA were younger with less comorbid conditions across all years compared to medically managed patients. Conversely, in patients who were medically managed, a greater proportional increase in the non-cardiac comorbidities was observed. The prevalence of renal failure increased from 10.8% to 34.0% and prevalence of dementia increased from 8.9% to 15.4% during the study period.

**Table 2:** Demographics of patients receiving coronary angiogram for alternate year included in the study, from 2004–2014.

Number of Cases	2004	2006	2008	2010	2012	2014
	195,071	217,748	221,160	228,890	251,015	265,300
Age (year), Median IRQ	66 (56–76)	65 (55–76)	65 (55–76)	65 (55–75)	65 (56–75)	66 (56–75)
Men %	60.1%	61.5%	61.0%	61.7%	62.1%	62.5%
Ethnicity						
White	56.4%	55.8%	59.4%	65.4%	70.8%	71.0%
Black	7.3%	7.1%	7.8%	10.6%	10.7%	10.6%
Hispanic	4.8%	5.8%	5.3%	6.4%	7.0%	7.4%
Asian/Pacific Islander	1.1%	1.1%	1.6%	1.7%	1.8%	2.0%
Native American	0.2%	0.3%	0.9%	0.8%	0.6%	0.5%
Other	2.0%	2.7%	3.1%	2.1%	3.3%	2.9%
Missing Race	28.2%	27.2%	21.8%	13.1%	5.8%	5.5%
Weekend admission	24.0%	24.2%	25.0%	25.7%	25.0%	25.6%
Primary expected payer						
Medicare	53.3%	52.2%	51.7%	51.8%	54.7%	55.0%
Medicaid	5.7%	5.7%	6.1%	7.2%	6.9%	8.8%
Private Insurance	32.8%	32.8%	32.3%	30.7%	27.5%	27.8%
Self-pay	5.0%	5.4%	6.0%	6.8%	7.0%	5.2%
No charge	0.6%	0.7%	0.6%	0.5%	0.6%	0.6%
other	2.6%	3.0%	3.3%	3.0%	3.2%	2.5%
Median Household Income (percentile)						
0–25^th^	26.4%	26.4%	28.3%	31.1%	32.1%	30.3%
26–50^th^	28.1%	26.6%	29.4%	28.0%	26.5%	29.5%
51–75^th^	23.3%	25.1%	22.9%	23.2%	22.8%	22.5%
76–100^th^	22.2%	21.9%	19.3%	17.7%	18.6%	17.7%
Comorbidities, %						
Dyslipidaemia	46.7%	50.4%	54.3%	58.7%	61.5%	61.8%
Smoking	27.8%	32.0%	35.1%	39.1%	42.7%	46.7%
Previous AMI	7.8%	8.1%	8.5%	9.7%	10.8%	11.4%
History of IHD	80.8%	80.6%	82.0%	82.3%	82.3%	81.0%
Previous PCI	7.3%	8.9%	10.0%	12.2%	13.8%	15.3%
Previous CABG	5.6%	5.3%	5.4%	5.6%	6.2%	6.5%
Previous CVA	No data	No data	2.8%	4.2%	4.7%	5.5%
Family history of CAD	5.0%	5.9%	6.8%	9.4%	9.3%	10.0%
Valvular heart disease	0.2%	0.2%	0.2%	0.1%	0.1%	0.1%
Peripheral vascular disease	9.7%	10.6%	12.1%	11.5%	12.8%	13.1%
Use of assist devise or IABP	4.1%	4.0%	4.5%	4.2%	4.3%	3.9%
Shock	1.8%	2.1%	2.5%	2.7%	2.9%	3.2%
AIDS	0.1%	0.13%	0.11%	0.14%	0.12%	0.14%
Alcohol abuse	2.3%	2.7%	2.8%	3.2%	3.2%	3.4%
Deficiency anaemias	8.9%	10.0%	13.5%	13.9%	15.0%	14.9%
Chronic Blood loss anaemia	1.1%	1.1%	1.0%	0.7%	0.7%	0.6%
RA/collagen vascular						
Diseases	1.6%	1.9%	2.2%	2.2%	2.4%	2.6%
Congestive heart failure	0.7%	0.6%	0.4%	0.6%	0.5%	0.4%
Chronic pulmonary disease	18.7%	20.2%	20.1%	19.8%	21.4%	21.8%
Coagulopathy	3.1%	3.2%	3.6%	4.5%	5.0%	5.2%
Depression	4.0%	5.0%	6.3%	7.2%	8.3%	8.7%
Diabetes	26.7%	27.8%	29.7%	30.6%	32.8%	33.2%
Diabetes with complications	4.5%	4.9%	5.6%	6.1%	7.1%	7.7%
Drug abuse	1.3%	1.9%	1.9%	2.2%	2.5%	2.8%
Hypertension	62.0%	66.2%	70.0%	72.6%	75.5%	77.2%
Hypothyroidism	6.9%	7.4%	9.1%	9.5%	10.8%	11.5%
Liver disease	0.7%	0.8%	1.1%	1.2%	1.5%	1.8%
Lymphomas	0.4%	0.4%	0.5%	0.4%	0.5%	0.5%
Fluid and electrolyte disturbances	10.9%	12.8%	15.3%	16.5%	18.4%	20.1%
Other neurological disorders	2.8%	3.3%	4.0%	3.9%	4.5%	4.9%
Obesity	8.9%	9.9%	13.7%	14.6%	18.1%	19.9%
Paralysis	0.9%	1.0%	1.4%	1.2%	1.2%	1.3%
Psychoses	1.2%	1.2%	2.0%	2.0%	2.3%	2.5%
Pulmonary circulation disorder	0.03%	0.03%	0.1%	0.1%	0.07%	0.07%
Renal failure (chronic)	6.4%	12.7%	14.4%	16.5%	17.8%	19.1%
Peptic ulcer disease	0.07%	0.06%	0.04%	0.02%	0.03%	0.02%
Weight loss	0.6%	0.9%	1.4%	1.6%	2.1%	2.0%
Solid tumour without Mets	0.9%	1.0%	1.1%	1.0%	1.1%	1.1%
Metastatic cancer	0.3%	0.4%	0.4%	0.4%	0.4%	0.4%
Dementia	1.7%	1.9%	2.4%	2.6%	3.1%	2.9%
Comorbidities - Charlson Comorbidity Index						
0	38.4%	38.4%	35.6%	34.2%	31.6%	30.2%
1	33.9%	33.9%	34.0%	32.9%	32.1%	31.6%
2	18.4%	18.2%	19.0%	19.8%	20.4%	21.0%
≥3	9.3%	9.3%	11.4%	13.1%	15.9%	17.2%
Hospital bed size						
Small	8.1%	8.7%	7.5%	8.6%	8.4%	12.7%
Medium	19.6%	21.4%	20.0%	18.8%	24.3%	28.9%
Large	72.3%	69.9%	72.5%	72.6%	67.3%	58.3%
Hospital Region						
Northeast	22.0%	18.8%	16.4%	17.4%	16.7%	17.0%
Midwest	24.1%	22.2%	25.3%	26.1%	23.8%	24.1%
South	39.1%	44.3%	42.5%%	38.8%	42.9%	42.2%
West	14.8%	14.7%	15.7%	17.7%	16.6%	16.7%
Location/Teaching status						
Rural	5.2%	4.4%	7.8%	10.1%	6.8%	5.7%
Urban-non teaching	38.2%	39.6%	40.5%	40.3%	38.2%	27.5%
Urban- teaching	56.6%	56.0%	51.7%	49.6%	55.0%	66.8%
Length of stay, Median (IQR)	4(2–7)	3 (2–6)	3 (2–6)	3 (2–6)	3 (2–6)	3 (2–6)
Total charge, $, Median (IQR)	39109 (24353–64456)	44175 (27482–71955)	49439 (31005–81857)	53105 (33083–88071)	57393 (36054–94376)	64487 (40537–105371)
Bleeding complications	11.5%	11.3%	11.7%	10.5%	10.0%	8.5%
Vascular complications	1.4%	1.5%	1.5%	1.1%	1.1%	1.1%
Cardiac complication	2.5%	2.3%	2.6%	2.0%	1.8%	1.9%
In-hospital mortality	2.2%	2.0%	2.0%	1.8%	1.8%	1.9%

**Table 3:** Demographics of patients not receiving coronary angiogram for alternate year included in the study, from 2004–2014.

Number of Cases	2004	2006	2008	2010	2012	2014
	207,084	184,979	182,214	158,144	148,900	142,455
Age (year), Median IRQ	75(61–84)	76(62–84)	77 (64–86)	77 (64–86)	77 (64–86)	76 (64–86)
Men %	49.0%	49.4%	49.3%	49.3%	50.0%	51.2%
Ethnicity						
White	56.6%	57.2%	62.7%	65.5%	71.1%	70.9%
Black	9.4%	8.7%	8.5%	12.4%	11.3%	12.1%
Hispanic	5.8%	6.9%	5.5%	6.2%	7.4%	7.5%
Asian/Pacific Islander	1.5%	1.7%	1.8%	2.3%	2.3%	2.1%
Native American	0.3%	0.4%	0.5%	0.6%	0.5%	0.5%
Other	1.9%	1.5%	2.3%	2.1%	2.6%	2.6%
Missing Race	24.5%	23.6%	18.6%	10.8%	4.7%	4.3%
Weekend admission	26.6%	26.0%	27.2%	27.3%	26.5%	26.9%
Primary expected payer, %						
Medicare	68.6%	70.5%	72.0%	72.9%	75.1%	74.4%
Medicaid	6.0%	5.2%	5.5%	5.8%	6.1%	7.5%
Private Insurance	19.4%	18.2%	17.1%	15.1%	13.0%	13.3%
Self-pay	4.0%	4.0%	3.1%	3.7%	3.6%	2.8%
No charge	0.3%	0.3%	0.2%	0.4%	0.3%	0.3%
other	1.8%	1.8%	2.1%	2.0%	1.9%	1.7%
Median Household Income (percentile)						
0–25^th^	31.1%	30.1%	28.9%	30.5%	31.7%	30.3%
26–50^th^	27.8%	26.7%	28.5%	26.9%	25.6%	27.0%
51–75^th^	21.2%	22.3%	21.8%	23.2%	22.6%	22.7%
76–100^th^	20.0%	20.9%	20.8%	19.4%	20.1%	20.0%
Comorbidities, %						
Dyslipidaemia	28.6%	32.3%	35.5%	40.4%	44.9%	46.8%
Smoking	15.3%	17.9%	19.3%	24.1%	27.9%	33.9%
Previous AMI	7.3%	7.8%	8.4%	10.6%	11.7%	11.9%
History of IHD	36.2%	38.7%	42.1%	44.7%	47.7%	47.9%
Previous PCI	4.6%	5.1%	6.3%	8.6%	11.0%	12.4%
Previous CABG	8.4%	8.6%	8.9%	10.7%	11.9%	12.5%
Previous CVA	No data	No data	3.8%	6.5%	7.6%	8.6%
Family history of CAD	2.6%	2.8%	2.9%	3.7%	3.9%	4.5%
Valvular heart disease	0.3%	0.4%	0.4%	0.5%	0.5%	0.3%
Peripheral vascular disease	8.4%	9.4%	11.3%	12.5%	13.8%	14.6%
Use of assist devise or IABP	0.3%	0.5%	0.5%	0.4%	0.7%	0.9%
Shock	1.6%	1.7%	1.7%	2.2%	2.9%	3.4%
AIDS	0.14%	0.08%	0.12%	0.15%	0.11%	0.15%
Alcohol abuse	2.0%	2.2%	2.4%	2.4%	2.7%	2.9%
Deficiency anaemias	13.9%	15.7%	20.9%	22.7%	25.3%	25.1%
Chronic Blood loss anaemia	1.9%	2.0%	1.6%	1.4%	1.3%	1.1%
RA/collagen vascular diseases	1.8%	2.0%	2.3%	2.7%	2.8%	2.9%
Congestive heart failure	1.3%	1.0%	1.3%	1.6%	1.2%	1.2%
Chronic pulmonary disease	23.5%	24.9%	24.9%	24.9%	26.7%	27.0%
Coagulopathy	2.8%	3.2%	3.7%	4.6%	6.1%	6.7%
Depression	5.4%	6.3%	7.8%	8.6%	9.6%	10.1%
Diabetes	27.2%	28.4%	29.7%	31.1%	32.7%	33.2%
Diabetes with complications	5.9%	6.4%	7.3%	7.6%	8.5%	9.7%
Drug abuse	1.3%	1.6%	1.5%	1.8%	2.0%	2.6%
Hypertension	59.1%	63.5%	67.6%	71.4%	74.5%	76.4%
Hypothyroidism	9.0%	10.3%	12.7%	13.7%	15.7%	16.0%
Liver disease	1.1%	1.2%	1.2%	1.5%	1.8%	2.2%
Lymphomas	0.5%	0.5%	0.7%	0.7%	0.8%	0.8%
Fluid and electrolyte disturbances	19.3%	21.5%	25.1%	26.1%	29.1%	30.8%
Other neurological disorders	7.0%	7.9%	9.6%	10.0%	10.4%	10.2%
Obesity	6.1%	6.8%	8.5%	9.2%	11.7%	13.6%
Paralysis	2.2%	2.3%	3.1%	2.8%	2.7%	2.7%
Psychoses	1.9%	2.1%	2.7%	3.1%	3.3%	3.5%
Pulmonary circulation disorder	0.06%	0.06%	0.2%	0.2%	0.2%	0.2%
Renal failure (chronic)	10.8%	21.1%	24.5%	29.1%	32.4%	34.0%
Peptic ulcer disease	0.07%	0.05%	0.04%	0.06%	0.02%	0.03%
Weight loss	1.8%	1.9%	3.1%	3.8%	4.6%	4.6%
Solid tumour without Mets	1.7%	1.8%	2.2%	2.3%	2.2%	2.2%
Metastatic cancer	1.2%	1.3%	1.6%	1.5%	1.6%	1.8%
Dementia	8.9%	10.8%	13.2%	14.9%	15.9%	15.4%
Comorbidities - Charlson Comorbidity Index						
0	28.4%	26.8%	23.6%	21.0%	19.0%	17.6%
1	32.8%	33.6%	32.4%	30.8%	29.5%	28.7%
2	23.2%	23.9%	24.8%	25.3%	25.5%	25.6%
≥3	15.6%	15.7%	19.2%	22.9%	26.0%	28.1%
Hospital bed size						
Small	15.9%	16.7%	16.0%	13.7%	16.4%	22.3%
Medium	28.6%	27.9%	26.5%	27.2%	29.1%	31.3%
Large	55.5%	55.4%	57.5%	59.1%	54.5%	46.4%
Hospital Region						
Northeast	26.9%	28.6%	24.5%	24.9%	25.5%	24.7%
Midwest	21.5%	19.0%	21.1%	21.8%	20.2%	20.4%
South	38.1%	38.3%	39.5%	38.5%	37.6%	37.9%
West	13.5%	14.1%	14.9%	14.8%	16.7%	17.0%
Location/Teaching status						
Rural	21.6%	19.6%	17.9%	17.9%	16.0%	13.7%
Urban-non teaching	50.5%	45.6%	50.1%	48.1%	45.0%	32.8%
Urban- teaching	27.9%	34.8%	32.0%	34.0%	39.0%	53.5%
Length of stay, Median (IQR)	3(2–6)	3 (2–6)	3 (2–6)	3 (2–6)	3 (2–6)	3 (2–6)
Total charge,$, Median (IQR)	13153 (7219–25739)	15770 (8585–30362)	18276 (10103–33945)	18830 (10586–34652)	21862 (12141–40742)	24223 (13606–45146)
Bleeding complications	11.2%	11.7%	12.7%	12.2%	11.9%	10.6%
Vascular complications	0.3%	0.3%	0.3%	0.3%	0.4%	0.5%
Cardiac complication	0.3%	0.5%	0.4%	0.4%	0.5%	0.6%
In-hospital mortality	6.8%	6.6%	6.7%	6.4%	7.0%	6.7%

Crude in-hospital mortality for patients receiving CA decreased from 2.2% in 2004 to 1.9% (P_trend_ < 0.001) in 2014; mortality rates remained fairly stable in the medically managed cohort (6.8% in 2004 to 6.7% in 2014; P_trend_ = 0.83).

**Figure 3:**
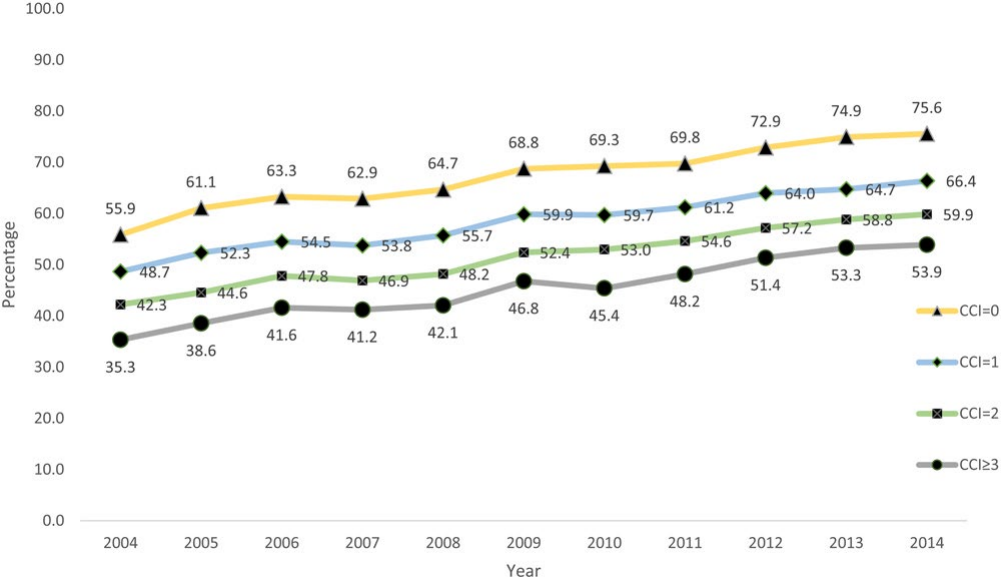
Proportions of patients receiving CA according to their comorbidity burden as defined per Charlson from 2004–2014.

**Figure 4:**
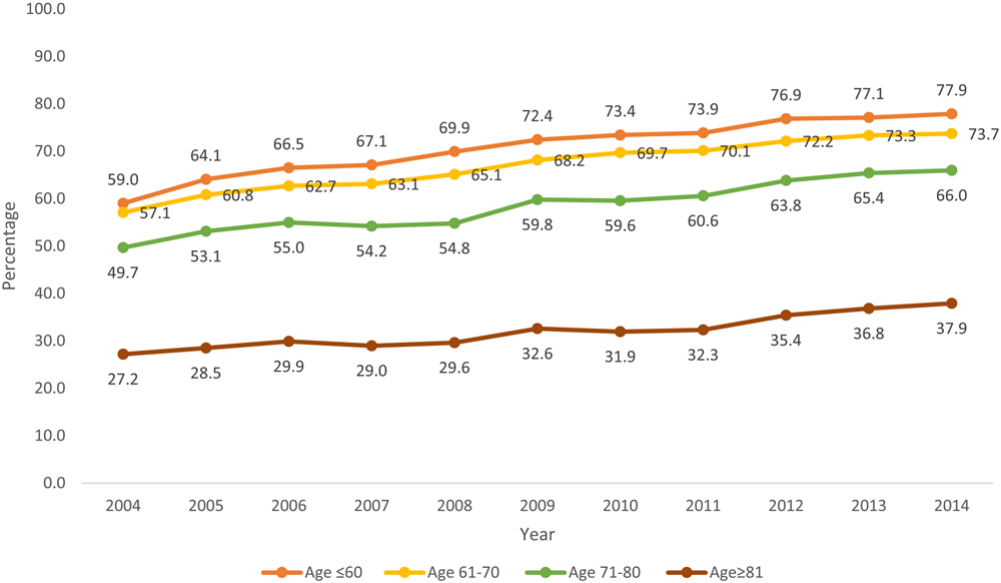
Proportions of patients receiving coronary angiography according to their age category from 2004–2014.

**Figure 5:**
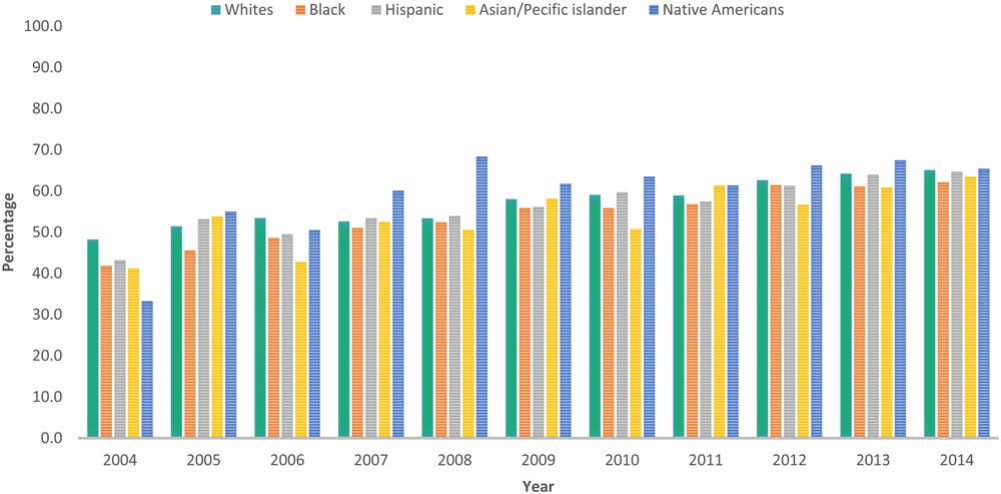
Porpotions of patients receiving coronary angiography according to their Ethnicity from 2004–2014.

**Figure 6:**
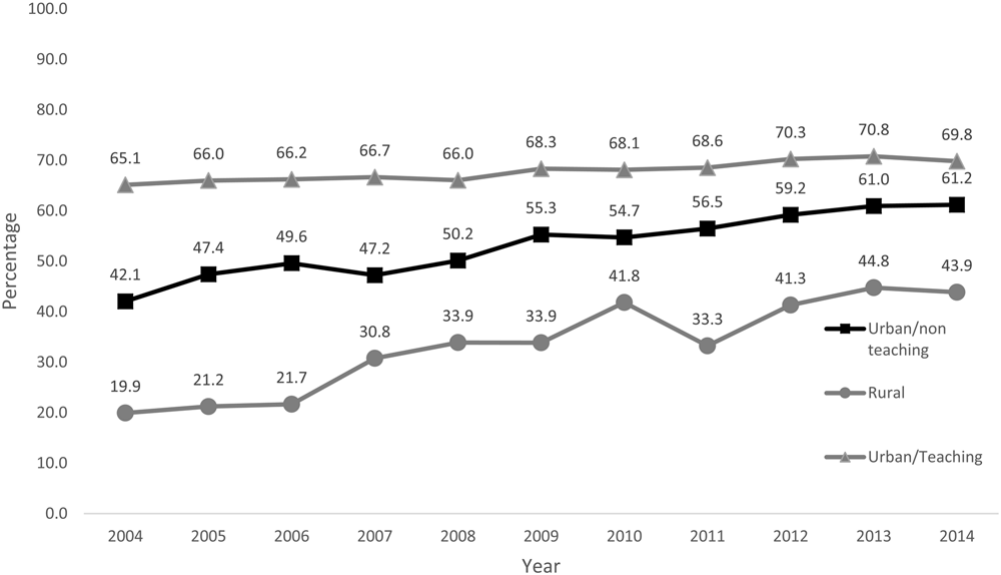
Porpotions of patients receiving coronary angiography according to the teaching status and location of hospital from 2004–2014.

Temporal trends in comorbidity burden of patients receiving CA as defined per Charlson score are shown in Fig. 3. An increase in the use of CA was noted across all four categories of Charlson score albeit with the lowest uptake in patients with highest comorbidity burden. From 2004 to 2014, the use of CA increased from 55.9% to 75.6% in patients with no comorbidity (CCI = 0), from 48.7% to 66.4% in CCI = 1, from 42.3% to 59.9% in CCI = 2 and 35.3% to 53.9% in CCI ≥ 3 category. Similar disparities were observed in use of CA when patients were stratified according to age group, ethnicity and hospital location/ teaching status (Figs 4–6**)**. Patients aged ≤ 60 years showed a higher proportional increase in utilization of CA (59.0% to 77.9%) compared to patients aged ≥ 81(27.2% to 37.9%). The adoption of CA was lower in African Americans (41.8% to 62.1%, P_trend_ < 0.001) and Asians (41.2% to 63.5%, P_trend_ < 0.001) compared to Whites. Institutional differences in practice are depicted in, while there has been a steady increase in use of CA in teaching hospitals (65.1% to 69.8%, P_trend_ < 0.001), a greater increase was noted in patients admitted to urban non-teaching (42.1–64.2%, P_trend_ < 0.001) and rural hospitals (19.9% to 43.9%, P_trend_ < 0.001). Finally, women were less likely to receive CA compared to men (58.9% vs 69.4%, P_trend_ < 0.001) (Supplementary Figure 1).

### Clinical and Institution predictors of Coronary Angiography

The independent predictors for use of CA following adjustment for differences in baseline covariates are shown in Table 4. Patient characteristics known to be related to adverse outcomes in NSTEACS such as increasing age (OR 0.96 95%CI 0.960–0.961), prior history of CABG (OR 0.35 95%CI 0.35–0.36), prior PCI (OR 0.84 95%CI 0.83–0.86), history of diabetes (OR 0.88 95%CI 0.87–0.89), diabetes with complications (OR 0.85 95%CI 0.83–0.87) and previous history of AMI (OR 0.65 95%CI 0.64–0.67) had a strong inverse relationship with receipt of CA. The independent predictors of CA use included the history of smoking (OR 1.17 95%CI 1.16–1.19), dyslipidemia (OR 1.39 95%CI 1.37–1.40), and history of IHD (OR 6.21 95%CI 6.13–6.29). Patients treated at large bed or teaching hospital had approximately a 3 and 5-fold increase in odds of receiving CA (large hospital bed size (OR 3.05 95%CI 2.99–3.11) and urban hospital teaching status (OR 5.51 95%CI 5.39–5.62)) compared to small bed size hospital and rural non-teaching hospital respectively.

**Table 4:** Independent Variables associated with receipt of angiography after excluding for in-hospital mortality.

Predictors	Odds Ratio	95% Confidence Interval	P Value
Age	0.96	0.960–0.961	<0.001
Weekend admission	0.92	0.91–93	<0.001
Female	0.91	0.90–0.92	<0.001
AIDS	0.79	0.67–0.93	<0.001
Alcohol abuse	0.88	0.85–0.91	<0.001
Deficiency anaemias	0.82	0.81–0.83	<0.001
Chronic Blood loss anaemia	0.72	0.68–0.76	<0.001
Congestive heart failure	0.53	0.49–0.57	<0.001
Chronic pulmonary disease	0.85	0.84–0.86	<0.001
Coagulopathy	1.12	1.08–1.15	<0.001
Depression	0.86	0.84–0.86	0.009
Diabetes	0.88	0.87–0.89	<0.001
Diabetes with complications	0.85	0.83–0.87	<0.001
Drug abuse	0.66	0.63–0.68	<0.001
Hypertension	1.02	1.00–1.03	0.001
Hypothyroidism	0.92	0.91–0.94	<0.001
Liver disease	0.76	0.72–0.80	<0.001
Fluid and electrolyte disturbances	0.85	0.83–0.86	<0.001
Other neurological disorders	0.79	0.76–0.81	<0.001
Obesity	1.09	1.07–1.11	<0.001
Paralysis	0.61	0.58–0.64	<0.001
Psychoses	0.67	0.64–0.70	<0.001
Renal failure (chronic)	0.66	0.65–0.67	<0.001
Weight loss	0.80	0.77–0.83	<0.001
Solid tumour without Mets	0.67	0.64–0.70	<0.001
Metastatic cancer	0.33	0.31–0.0.35	<0.001
Dementia	0.32	0.31–0.33	<0.001
Dyslipidaemia	1.39	1.37–1.40	<0.001
Smoking	1.17	1.16–1.19	<0.001
Previous AMI	0.65	0.64–0.67	<0.001
History of IHD	6.21	6.13–6.29	<0.001
Previous PCI	0.84	0.83–0.86	<0.001
Previous CABG	0.35	0.35–0.36	<0.001
Previous CVA	0.82	0.79–0.84	<0.001
Family history of CAD	1.30	1.27–1.34	<0.001
Peripheral vascular disease	1.12	1.10–1.14	<0.001
Shock	2.16	2.06–2.26	<0.001
Hospital bed size(Ref small)			
Medium	1.56	1.52–1.59	<0.001
Large	3.05	2.99–3.11	<0.001
Location/Teaching status (Ref Rural)			
Urban-non teaching	2.58	2.53–2.63	<0.001
Urban- teaching	5.51	5.39–5.62	<0.001

### Use of angiography and clinical outcomes

Association between the use of CA and in-hospital outcomes are reported in Table 5. In our multivariable analysis, CA was associated with a significantly decreased odds of in-hospital death (OR 0.38 95%CI 0.36–0.40) and a significant increase in the odds of cardiac complications (OR 4.77 95%CI 3.88–5.87), major bleeding (OR 1.23 95%CI 1.16–1.31), and vascular complications (OR 3.96 95%CI 3.09–5.07) after adjusting for age, sex, year of procedure, 29 Elixhasuer comorbidities, ethnicity, median income, weekend/weekday admission, cardiovascular risk factors and hospital characteristics.

**Table 5:** Association between use of coronary angiography and in hospital clinical outcomes.

Clinical outcome	Odds Ratio	95% Confidence Interval	P value
In hospital mortality	0.38	0.36–0.40	<0.001
Cardiac Complications	4.77	3.88–5.87	<0.001
Major Bleeding	1.23	1.16–1.31	<0.001
Vascular Complications	3.96	3.09–5.07	<0.001

In the subgroup analysis of patients age ≧65 yrs and age <65 yrs, White race and non-white race, large, medium, and small size hospital and CCI ≧ 3 or <3, receipt of CA was associated with lower odds of in-hospital mortality in patients age <65, Whites, large hospitals and CCI <3 compared to patients age ≧65 yrs, non-whites, small hospital and CCI ≧ 3 respectively (Supplementary Figure 2). In the propensity matched analysis, use of CA remained significantly associated with reduced risk of in-hospital mortality and increased bleeding, vascular and cardiac complications (Supplementary Table 4).

## Discussion

Coronary angiography remains the gold standard for the evaluation of NSTEACS. In this analysis of over 4.3 million patients admitted with a diagnosis of NSTEACS in the US, we report an increase in the uptake of CA over an 11-year period. Our results demonstrate a paradigm shift in the demographics, comorbidity and risk profile of patient presenting with NSTEACS resulting in the use of CA in a more complex, elderly and multimorbid cohort. Despite the increased adoption of CA, there were significant disparities at both a patient and institutional level in CA practices with particularly slower adoption in use of CA in older, women, higher comorbidity burden, African Americans cohorts and those admitted to rural or non-teaching hospitals. In addition, we observed a strong risk-treatment paradox, in that clinical groups of patients who most likely benefit from the receipt of CA, were least likely to receive it.

Our results demonstrate that the clinical spectrum, baseline characteristics and comorbidity status of patients presenting with NSTEACS have changed significantly over the past decade reflecting the ageing demographics in the US. Patients requiring CA are older, more complex and burdened with multiple comorbidities. More importantly, we observed significant heterogeneity in the utilization of CA in different patient groups stratified according to sex, age, ethnicity and hospital characteristics. A higher proportional increase in the utilization of CA was noted in young patients aged ≤60 years compared to elderly patients (aged ≥81) despite a progressive increase in the average age of NSTEACS population. The inequalities in use of CA were also evident in women and African Americans wherein adoption of CA has been particularly slower in comparison to men and Native Americans respectively. African Americans and Asians were almost 30% less likely to receive CA. Teaching hospital status was associated with a higher use of CA compared to rural hospitals despite the expansion of angiography services in rural hospitals^16^.

The delay in uniform adoption of invasive coronary approach across the whole spectrum of NSTEACS patients may be related to a complex web of underlying factors including local practice style, service availability and inequalities in uniform health coverage by the insurance-based system. It is important to note that the under-utilisation of both invasive and medical therapies in women and the elderly have been widely described which in part has been related to the increased perception of adverse outcomes in women and older patients^4,17,18^. Increase knowledge and understanding of important factors which influence clinician’s decision-making about use of CA is required to ensure a uniform and effective use of invasive CA in this underserved group of patients.

Our analysis allows for the study of the temporal changes in the clinical characteristics and associated comorbidities of the patients receiving CA compared to those medically managed. Previous studies have mainly reported on the cardiovascular comorbid burden of NSTEACS patients such history of hypertension, dyslipidaemias, smoking, and diabetes^4,5,7,19,20^. However, the granularity of comorbidity data in NIS facilitates the study of both cardiac and non-cardiac comorbidities in decision making in much greater detail. Our analysis illustrates that non-cardiac comorbid burden has increased considerably in patients with NSTEACS over the last decade. We observed a significantly higher prevalence of non-cardiac comorbidities such dementia, chronic obstructive airway disease, renal disease and cancer in patients not receiving coronary angiography. We also observed significant disparities in selection for CA and global measures for the severity of comorbidity burden. Utilization of CA remained lower in patients with a higher Charlson score category (CCI ≥ 3) compared to no comorbidity (CCI = 0) group throughout the study time period. There is a paucity of data on the utilisation of invasive coronary management in patients with multimorbidity as these patients are often excluded from randomised control trials^21^. It is conceivable that treating physicians may adopt a more conservative approach in older, frailer and multimorbid patients due to the perceived increased risk of adverse events. However, previous studies have shown that impact on mortality with invasive therapies for ACS is not attenuated with age^18^ and patients with higher comorbidities may have even greater gains from an invasive approach^22,23^. Therefore, age alone or presence of comorbidities should not deter the physician from performing CA. Women have often been denied an early invasive approach^24,25^ but recent data from Ontario, Canada showed that women had worse outcomes after undergoing early CA after NSTEACS^26^, when compared to men. Women had more bleeding complications after undergoing CA but it was also seen that women were less likely than men to undergo any revascularization even after undergoing CA. Younger women were less likely to undergo CA in this population, there were no noted sex-differences in outcomes in those receiving medical management rather than an invasive approach. These observational data may bias management of female patients, where CA continues to underutilize in women.

Current guidelines advocate a risk-based approach for offering an invasive strategy in the setting of NSTEACS which includes several parameters such as age, history of renal insufficiency, prior history of CABG or PCI and presence of coronary disease risk factors such as diabetes^10,13^. Our study shows that patient features which are known to be associated with increased risk of adverse events in NSTEACS such as age, diabetes with complications, prior history of CABG, PCI or AMI actually have a strong inverse relationship with receipt of CA. In a previous analysis of the CRUSADE registry, Cohen *et al*. reported that patients with the greatest possibility of having severe coronary artery disease were least likely to have CA^5^. However, patients with prior CABG, severe comorbidities and advanced age were excluded from this analysis. With this analysis, we add to this literature by using granular data from both cardiac and non-cardiac comorbidities, older age, racial and institutional factors thus representing a truly real-world population elucidating a persistent treatment-risk paradox.

The strengths of our study findings arise from our use of comprehensive, unselected, national records that are derived from an obligatory administrative database which are representative of true real-world practice. The granularity of comorbidity data, diversity in geographic, racial and hospital characteristic information within the NIS dataset allowed us to study the disparities in CA practices.

Nevertheless, our work must be interpreted within the context of certain limitations. First, our work is observational in nature and the possibility of unmeasured confounders cannot be ruled out. Secondly, important clinical information such as medication history, frailty, ECG and cardiac biomarker information is not captured within the NIS database. Cardiac biomarkers, ECG changes, and hemodynamic parameters are important for risk stratification and may influence a physician’s decision on whether to adopt an invasive approach^27^. The information regarding onsite facility to perform angiography is not available in the database which may have limited the estimation of utilisation of CA. Finally, as with any administrative database, there is a potential for coding error for diagnoses or procedure codes.

## Conclusion

In conclusion, this analysis of NIS dataset found that patients admitted with NSTEACS undergoing CA have significantly changed over the past decade towards a higher risk profile, with increasing number of elderly and comorbid patients undergoing CA. There were, however, groups of patients in whom the rate of increase in access to CA was lower: elderly, women, African Americans, Asians and those being admitted to rural small hospitals. Although utilization of CA was associated with decreased odds of in-hospital mortality, there remains a risk-treatment paradox, where patients more likely to benefit from an invasive management strategy were least likely to receive it. Our study provides information for healthcare providers to develop strategies designed to ensure fair and appropriate access to CA for patients admitted with NSTEACS who are likely to benefit from pharamcoinvasive management.

## Electronic supplementary material

supplementary material
