# Supplementary material for: Temporal trends and inequalities in coronary angiography utilization in the management of non-ST-Elevation acute coronary syndromes in the U.S
Source: Sci Rep. 2019 Jan 18;9:240. doi: 10.1038/s41598-018-36504-y (PMC6338770; doi:10.1038/s41598-018-36504-y)
Supplement: Supplementary file 1 — supplementary material [file 41598_2018_36504_MOESM1_ESM.docx]

**Temporal trends and inequalities in coronary angiography utilization in the management of non-ST-Elevation acute coronary syndromes in the U.S.**

**Short running title:** Use of coronary angiography in NSTEACS

Muhammad Rashid MBBS^a,b^, David L. Fischman MD^c^, Martha Gulati MD^d^ Khalid Tamman MD^e^, Jessica Potts MSc^a^, Chun Shing Kwok MBBS^a,b^, Joie Ensor PhD^a^, Ahmad Shoaib MD^a,b^, Hossam Mansour MD^f^, Azfar Zaman PhD^g^, Michael P. Savage MD^c^, Mamas A.Mamas^a,b^

1. Keele Cardiovascular Research Group, Centre for Prognosis Research, Institutes of Applied Clinical Science and Primary Care and Health Sciences, Keele University, Stoke-on-Trent, UK
2. Department of Cardiology, Royal Stoke Hospital, University Hospital North Midlands, Stoke-on-Trent, UK
3. Department of Medicine (Cardiology), Thomas Jefferson University Hospital, Philadelphia, Pennsylvania, United States
4. Division of Cardiology, University of Arizona, Phoenix, AZ
5. Department of Cardiology, International medical centre, Jeddah, Saudi Arabia
6. Department of Cardiology, Aswan University, Aswan, Egypt
7. Department of Cardiology, Freeman Hospital and Institute of Cellular Medicine, Newcastle University, Newcastle-upon-Tyne, UK.

Supplementary table 1: List of international classification of disease, Ninth Edition, clinical modification (ICD-9-CM) and clinical classification software codes used for identifying additional comorbidities

| **Comorbidities** | **Source** | **Codes** |
| --- | --- | --- |
| Dyslipidaemias | CCS | 53 |
| Coronary artery disease | ICD-9-CM | 414.00-414.07 |
| Family history of IHD | ICD-9-CM | V17.3 |
| Previous stroke or transient ischemic attack | ICD-9-CM | V12.54x |
| Previous CABG | ICD-9-CM | V45.81x |
| Previous PCI | ICD-9-CM | V45.82x |
| Cardiogenic shock | ICD-9-CM | 785.51 |
| Use of inotropic agents | ICD-9-CM | 00.17 |
| Use of inotropic assist device | ICD-9-CM | 376, 97.44 |
| Smoking | ICD-9-CM | V15.82, 305.1 |
| Dementia | ICD-9-CM | 290.xx,294.1x,294.2x,294.8,331.0,331.12,331.82,797 |

Supplementary Table 2: Deyo’s modification of Charlson’s co-morbidity index (CCI).

| Reported ICD-9 codes | Condition | Charlson score |
| --- | --- | --- |
| 412 | Previous myocardial infarction | 1 |
| 428 – 428.9 | Congestive heart failure | 1 |
| 433.9, 441 – 441.9, 785.4 V43.4 | Peripheral vascular disease | 1 |
| V12.54, 438.x | Previous cerebrovascular disease | 1 |
| 290 – 290.9 | Dementia | 1 |
| 490 – 496, 500 –505, 506.4 | Chronic pulmonary disease | 1 |
| 710.0, 710.1, 710.4, 714 – 714.2, 714.81, 725 | Rheumatologic disease | 1 |
| 531 – 534.9 | Peptic ulcer | 1 |
| 571.2, 571.5, 571.6, 571.4 –571.49 | Mild liver disease | 1 |
| 250 – 250.3, 250.7 | Diabetes | 1 |
| 250.4 – 250.6 | Diabetes with chronic complications | 2 |
| 344.1, 342 – 342.9 | Hemiplegia or paraplegia | 2 |
| 582 – 582.9, 583 – 583.7, 585, 586, 588 – 588.9 | Renal Disease | 2 |
| 140 – 172.9, 174 –195.8, 200 – 208.9 | Any malignancy including leukaemia and lymphoma | 2 |
| 572.2 – 572.8 | Moderate or severe liver disease | 3 |
| 196 – 199.1 | Metastatic solid tumour | 6 |
| 042 – 044.9 | AIDS | 6 |

Supplementary table 3: ICD-9-CM codes for post procedural complications

| **Post-procedural Complication** | **ICD-9-CM or CCS codes** |
| --- | --- |
| **Bleeding complication** |  |
| Gastrointestinal | CCS 153 |
| Unspecified haemorrhage | 459.0 |
| Retroperitoneal haemorrhage | 568.81, 998.1 |
| Intracranial haemorrhage | 430-432x |
| Post-op haemorrhage requiring transfusion | 99.0 (procedure) |
| Blood transfusion | V58.2 |
| **Vascular complications** |  |
| Vascular injury | 900-904, 998.2, 447, 868.04, 999.7 (diagnosis)  39.31, 39.41, 39.49, 39.52, 39.53, 39.56 - 39.59 39.79 (procedure) |
| **Cardiac complications** |  |
| Iatrogenic cardiac | 997.1 |
| Pericardial comp | 423.0, 423.3 (diagnosis) 47.0 (procedure) |
| Requiring CABG | 36.1x, 36.2, 36.31, 36.32, 36.9x |

Supplementary table 4**:** Propensity score matching analysis reporting average treatment effects (ATE) comparing coronary angiography versus no coronary angiography patients.

| CA vs No CA | Coefficient | 95% confidence interval | | P value |
| --- | --- | --- | --- | --- |
| In hospital death | -0.0346111 | -0.0361011 | -0.0331211 | <0.001 |
| Major cardiac complications | 0.0098089 | 0.0087383 | 0.0108796 | <0.001 |
| Major bleeding | 0.0048243 | 0.002299 | 0.0073495 | <0.001 |
| Vascular complications | 0.0062807 | 0.0053833 | 0.0071781 | <0.001 |

Supplementary figure 1: Proportions of patients receiving coronary angiography according to their gender category from 2004-2014


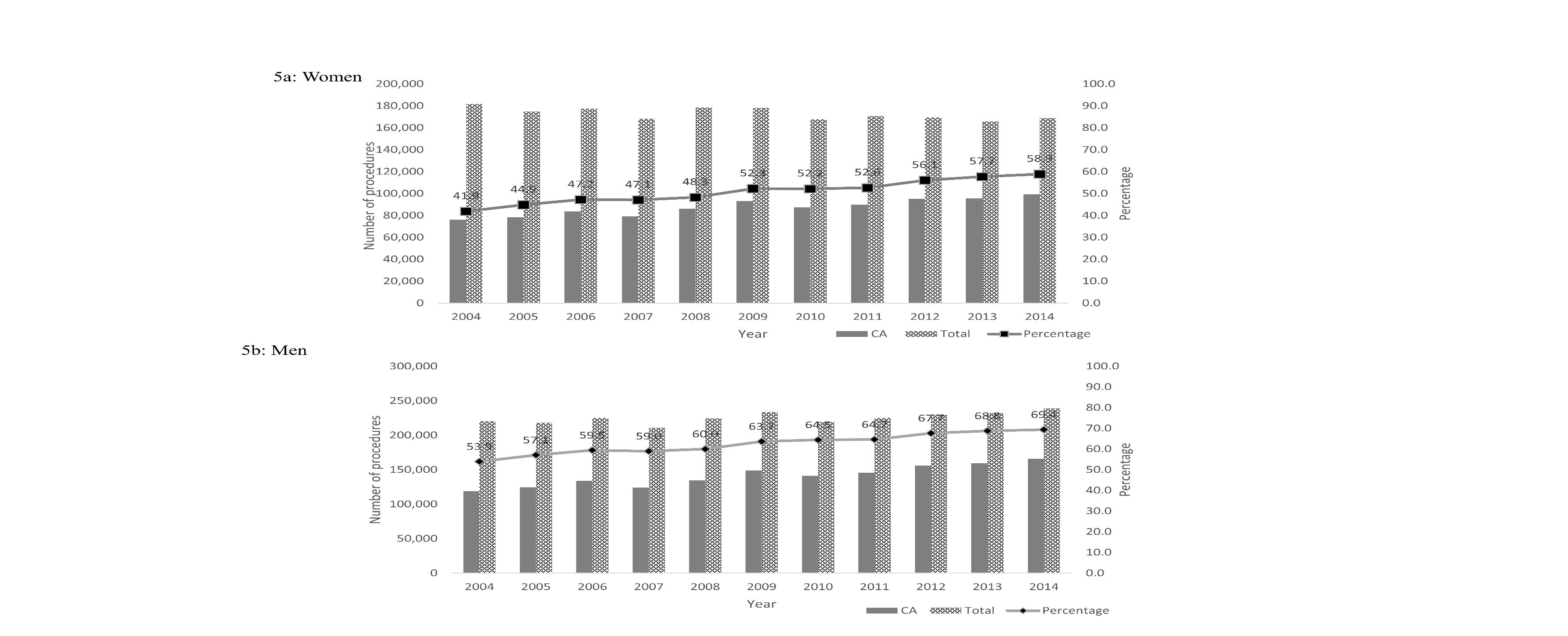


Supplementary figure 2: Subgroup analysis comparing association between use of coronary angiography and in-hospital mortality patients age ≧ 65yrs and age < 65yrs, White race and non-white race, hospital and CCI ≧ 3 or <3 and large, medium and small size hospitals
